# Factors Influencing Participation in Precision Health Research Among Diverse Individuals in South Carolina

**DOI:** 10.1007/s40615-026-02916-0

**Published:** 2026-04-23

**Authors:** Bernardica L. Brown, Paula S. Ramos, Lori Ann Ueberroth, Lee H. Moultrie, Marquetta L. Goodwine, Rachael J. Werner, Samiha T. Karim, Ingrid M. Wagner, Melissa A. Cunningham, Diane L. Kamen, Bethany J. Wolf, Caitlin G. Allen

**Affiliations:** 1 Department of Public Health Sciences, Medical University of South Carolina, Charleston, SC, USA; 2 Department of Medicine, Medical University of South Carolina, Charleston, SC, USA; 3 Department of Medicine, Emory University School of Medicine, Atlanta, GA, USA; 4 Lee H. Moultrie & Associates, North Charleston, SC, USA; 5 Gullah/Geechee Nation, St. Helena Island, SC, USA; 6 Department of Implementation Science, Wake Forest University School of Medicine, Winston-Salem, NC, USA

**Keywords:** Precision health, Research participation, Recruitment, Barriers, Facilitators, Qualitative research

## Abstract

**Background:**

Precision health research uses genomic, environmental, and lifestyle data to tailor disease prevention, health promotion, and treatment strategies. While it offers potential for improved diagnoses and personalized care, recruitment and retention remain challenging.

**Methods:**

The Precision rEsearCh pArticipatioN (PECAN) study used an exploratory qualitative design, guided by the socioecological model, to examine barriers and facilitators to participation in precision health research among diverse South Carolina residents. Ten focus groups were conducted with 23 participants, exploring individual, interpersonal, and community-level influences.

**Results:**

Barriers included mistrust in the medical system, limited awareness of research opportunities, and logistical issues such as transportation and scheduling. Facilitators included perceived health benefits, monetary incentives, ease of participation, and strong communication with the research team. Recommendations to address barriers included inclusive outreach, transparent communication about data use and participant expectations, leveraging provider relationships, and offering flexible scheduling and locations.

**Conclusion:**

The PECAN study highlights how mistrust, logistical barriers, and historical inequities—such as medical exploitation and limited rural research infrastructure—impede participation in precision health research. Facilitators like health benefits and effective communication can support engagement. Despite a small sample size, findings underscore the need for systemic change, including policy-level solutions to expand rural research infrastructure, enhance provider engagement, and ensure transparent data practices. These insights from rural and diverse communities emphasize the urgency of structural changes to promote equitable participation in precision health research.

## Introduction

Precision health research is a unique approach to healthcare that utilizes data from an individual’s genetics, environment, and lifestyle to develop personalized strategies for disease prevention and treatment [1]. However, this approach is limited by the ongoing challenge of recruitment. In addition to the objective barriers (such as time, technological literacy, language fluency) and subjective barriers to participation (including mistrust, fear, and stigma) shared among all research fields, specific challenges to patient recruitment in precision health research include a lack of familiarity with the precision health terminology and concerns about data privacy and security, as precision health research often requires the collection of more extensive personal data as compared to other forms of research [2–5].

There are multiple factors that impact recruitment across the individual, interpersonal, and community levels. The individual level of recruitment in precision health studies encompasses internal components such as beliefs and attitudes. It also includes personal circumstances such as education and socioeconomic status [6]. Common individual level barriers to recruitment include misunderstandings about the difference in terminology between “precision” medicine and “personalized” medicine. While these terms can be used interchangeably, some studies apply them in varying contexts, representing different outlooks on the future of medicine and assumptions about the role health care systems play [4, 7]. Furthermore, low health literacy, fear of genetic testing results, and the financial burden of precision health due to the cost of extensive data collection pose as barriers [8]. Individual level facilitators to recruitment in precision health research include familiarity of precision health research, level of education, altruism, compensation, and a high level of trust in the security of personal data [4, 9].

The interpersonal level involves the influence of participants’ social relationships on their decision to participate. These barriers to participation can often include distrust in medical research, poor communication between healthcare providers and patients, and a lack of cultural sensitivity from researchers [9]. Interpersonal factors that encourage recruitment often are in regard to trust. Facilitators include involving research staff who are representative of the research participants’ racial or ethnic group, and trust in the source of recruitment information such as a primary care provider, community organization, or the institution conducting the study [8, 9].

Community level factors focus on the participant’s interpersonal network. This involves awareness, culture, and some socioeconomic factors such as transportation and community resources. Common examples of community level barriers are stigma around research, awareness about research opportunities, limited access to information, and lack of community engagement [9]. Community level facilitators include strong community partnerships, community involvement in the design of the study, and convenience of participation in regard to support from workplaces and transportation to the study site [9, 10]. Legal factors such as inadequate privacy and anti-discrimination protections for research participants, along with practices by research sponsors that tolerate and entrench disparities, also serve as barriers that limit diversity and inclusion in genomic research [11].

Understanding the barriers and facilitators to individuals’ decision to participate in precision health research is essential for advancing the field. To ensure that clinical and translational research is generalizable and precision-based approaches can become attainable to everyone, research participants must reflect the diversity of the population. Historically marginalized groups continue to be underrepresented in research, perpetuating health disparities in treatment and outcomes across medicine. This underrepresentation may stem from unique cultural, socioeconomic, or physical barriers. Studies have explored the factors contributing to low participation among certain groups, with trust-based community relationships identified as a key factor in successful recruitment [12]. However, racial and ethnic groups in these studies are often treated as homogeneous, despite their cultural and genetic diversity, necessitating tailored participation strategies rather than a “one size fits all” approach.

South Carolina’s population is characterized by significant cultural and sociodemographic diversity. Given that individuals’ experiences vary across populations, geography, and cultures, it is essential to study these differences within their sociocultural context. Past research has not clearly established how sociocultural diversity influences South Carolinians’ beliefs and attitudes toward research participation and has not provided a comprehensive understanding of their perceptions regarding involvement in the emerging field of precision health research. The Precision rEsearCh pArticipatioN (PECAN) study sought to identify factors that positively and negatively influence perceptions about precision health research participation to identify actionable steps for improving understanding and reducing barriers to participation.

## Methods

### Research Design

PECAN used an exploratory qualitative study design to examine beliefs and perceptions, as well as identify facilitators and barriers to participation in precision health research. The study also aimed to identify recommendations for improvement in each domain. Researchers administered electronic surveys to community members (community leaders and social groups) for a 6-month period (between mid-December 2023 and mid-June 2024). A total of 308 individuals completed the survey. At the end of the survey, participants were asked if they would like to be contacted to participate in a peer discussion session, where they could provide insight and recommendations for participation in precision health research. Of the 101 survey participants who had expressed interest in joining a focus group, 93 were purposively selected and contacted based on the diversity of their survey responses and demographic characteristics to ensure that the sample was representative of the survey population. From this number, 23 participated in the scheduling process and completed a focus group session. The goal of these sessions was to involve community members more deeply in dialogue and decision-making regarding how to improve understanding and reduce barriers to participation in precision health research. This study was approved by the Institutional Review Board for Human Research (IRB) at the Medical University of South Carolina (Pro00126189).

### Participants

Demographic data of all focus group participants were collected from the initial survey, including age, sex, race/ethnicity, education level, lupus diagnosis, and prior knowledge of precision and personalized medicine. Age was categorized in about 9-year intervals, education was the highest degree earned, and understanding of precision and personalized medicine was reported on a five-point scale (not at all, slightly, somewhat, moderate, extremely). All participants provided informed consent and were informed that their participation was voluntary and that they could withdraw from the study at any time without penalty.

### Framework

To guide the overall methodology of this research, we applied a conceptual framework that considered the factors that influence decision-making processes. We were guided by a socioecological model that emphasizes the importance of integrating societal, community, interpersonal, and individual determinants to understand decisions to participate in precision health research in historically underrepresented groups (Supplementary Figure S1). This framework encompasses various levels of influence and the interactions between individuals and their environment. People’s lived experiences are shaped by factors such as race, ethnicity, socioeconomic status, geographic location, sexual orientation, gender identity, behavior, societal policies, and other characteristics [13]. As a result of this, people from different backgrounds may have different experiences. Due to these differences, it is important to study population preference and how it might impact diverse approaches to precision health research. We were also guided by the Health Belief Model, which posits that engagement in health behaviors is influenced by perceived disease susceptibility and severity, perceived barriers and benefits of action, cues to action, and self-efficacy [14].

We conducted 90-minute focus group sessions, which included the following topics: perceptions about research participation, unique aspects of participation in precision health research, opportunities to enhance research participation, and ways to recruit racial and ethnic minorities in research. Topics were informed by constructs from the socioecological model (Supplementary Figure S1), which emphasizes the interplay between individual, interpersonal, and community-level factors influencing health behaviors and research participation. To ensure that sessions flowed efficiently, an interview guide was created. The guide included 18 questions on the referenced topics, findings from the PECAN survey data previously acquired, and three case studies. The guide also included prompts to ensure session moderators addressed the individual, interpersonal, and community-level perspectives (Supplementary Box S1). The case studies were one paragraph in length and described a situation where an individual was presented with an opportunity to participate in a research study while faced with common barriers. The case studies described the individual’s response in the scenario and whether they chose to participate. Moderators asked participants to reflect on the individual’s decision in the scenario, whether they would have made a similar choice, and what factors might have persuaded them to participate; moderators served as facilitators when participants indicated they would not participate.

### Analytical Approach

**Fig. 1:**
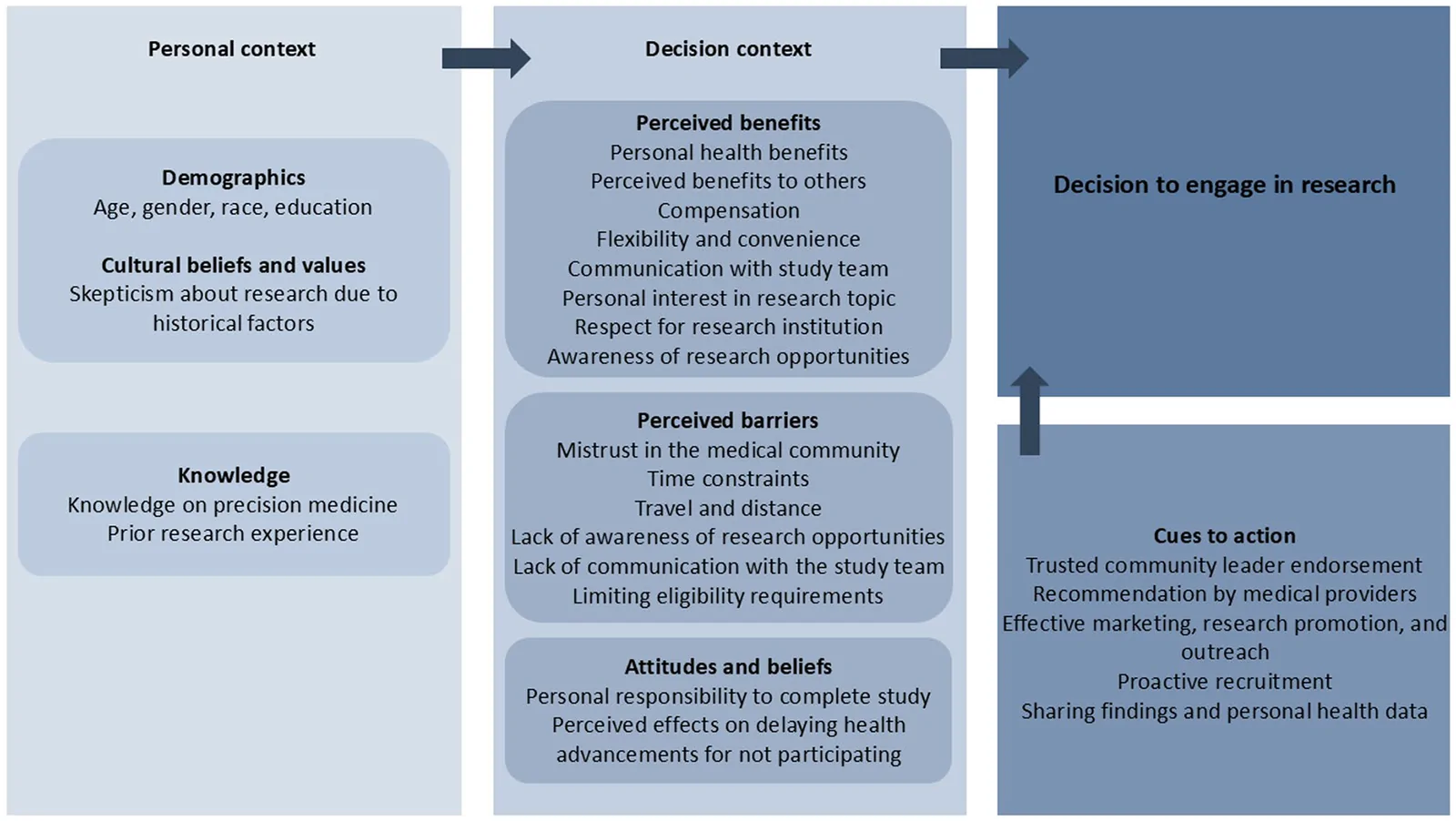
Health Belief Model Illustrating Results from Focus Groups

Quantitative survey results, including participants’ knowledge levels of precision health topics, age, race/ethnicity, and other demographic data, were integrated with focus group transcripts. Survey patterns, such as the percentage of participants in each demographic category, were compared with qualitative themes to help interpret findings and highlight underlying factors. Focus group sessions were recorded and transcribed verbatim with participant consent. Researchers created an initial codebook containing code names and definitions based on the key questions that the researcher desired to be answered by the focus groups. For example, the code labeled *individual-level facilitator* is defined as any reference to personal factors or conditions that encourage or enable participation in the research study, including knowledge, beliefs, perceptions, income, insurance, education, and age. Using this codebook, a transcript summary document was created for each focus group (Supplementary Box S2). Three independent team members (BB, PR, CA) created a document of this type for each focus group session to summarize the information and assign the collected data to relevant codes. These summaries allow for high-level analysis during ongoing data collection, facilitate codebook development, and reduce the number of new codes needed during data analysis. Each of the three independent summaries were then compiled by BB into one summary document and data matrix, which included codes and direct quotes from the focus groups. This approach allowed researchers to clearly identify common themes and assess saturation across participant responses, with thematic saturation achieved. The codes were used to construct a Health Belief Model (Fig. 1) that illustrates the results of the focus group sessions.

## Results

### Participants Characteristics

A total of 10 focus groups took place from September 2024 to October 2024. The final collection of data from focus groups was on Monday, October 21st, 2024. A total of 23 participants took part in the focus group sessions, with demographic characteristic detailed in Supplementary Table S1. The majority of participants were female (78%). Almost half of the participants, 43%, identified as African American. The age range of participants spanned between 18 and 75 years old. About 57% of participants had a 4-year college degree or higher, while the remaining percent did not complete this degree or preferred not to answer. About 26% of participants had a moderate or higher level of understanding of the term “precision medicine.” Additionally, about 52% had a moderate or higher understanding of “personalized medicine.” Of the 23 participants, 6 reported receiving a lupus diagnosis from a doctor.

### Thematic Analysis

The analysis identified several key barriers and facilitators influencing participation across various research study types, including basic research, clinical trials, and qualitative studies. These factors were coded at three levels: individual, interpersonal, and community. The individual level refers to personal challenges or factors that may impede or encourage study participation, such as knowledge, beliefs, perceptions, income, health insurance, education, and age. The interpersonal level involves challenges or facilitators that stem from interactions with others, including attitudes, cost, time, confidentiality, security, privacy, peer discrimination, and social support. Lastly, the community level captures broader contextual factors that may impede or encourage study participation, such as awareness, culture, geography, transportation, and partnership with research. Table 1 and Supplementary Tables S2 and S3 summarize the main barriers and facilitators to research participation found in this study.

The results of the thematic analysis of the focus groups are illustrated in the Health Belief Model shown in Fig. 1. This conceptual framework summarizes how the decision to participate in research is influenced by individual factors like participants’ sociodemographic characteristics, cultural beliefs, knowledge and previous experiences with research. It is also substantially influenced by the perceived barriers to and benefits from participating in research studies. The thematic analysis also showed that perceptions of individual responsibility to advance medical research contributes to their decision to engage in research. Cues to action includes people and strategies that can provide helpful cues to action for engaging individuals in research.

#### Individual-Level Barriers

Participants expressed eligibility requirements as a common barrier to participation, as detailed in Supplementary Figure S2, which outlines key barriers to participation and retention, their impact, and insights shared by focus group participants. Participants who found studies of interest were often deterred by unclear or poorly communicated eligibility criteria which disqualified them from moving forward. A participant said that “one of the barriers has been age. Well, they were looking for a certain segment of the population, but not until I was ready to apply did they say we are not seeking people of that particular age.”

#### Interpersonal-Level Barriers

The time it takes to participate in studies was a deterrent, as many participants stated that high-commitment studies were unfavorable and were often inconvenient. Participants stated the studies were often time consuming, and that they were “more likely to participate in a study where the commitment is low.”

Mistrust and privacy concerns emerged as significant barriers, with many participants expressing reluctance to participate in studies due to mistrust in the medical community. This hesitation was particularly evident in studies requiring the disclosure of health information. The Coronavirus (COVID-19) pandemic was mentioned in multiple sessions in regard to mistrust. One participant stated that after the COVID-19 pandemic “people just don’t trust automatically what their doctors are recommending or what the medical community is saying.”

Many African American participants expressed that negative historical events have deterred research participation in this population, especially among males. Some participants shared that their reason for participating in this study profoundly stemmed from a desire to enhance research by contributing data from this underrepresented group.

#### Community-Level Barriers

Many focus group participants mentioned geography and distance to the research site as a barrier, expressing that studies requiring in-person activity are inconvenient and often deter them from participating for reasons such as financial and occupational obligations, childcare, and transportation. Participants expressed that finding research opportunities online is challenging, limiting awareness and creating a significant barrier to their involvement. A participant stated, “you have to do deep searches and then their databases, like when you put in your information and they reach out to you, it’s still about stuff that doesn’t apply to you, so they gotta figure out a better system for that.”

#### Barriers to Retention

Participants stated that the largest barrier to retention in research is a lack of communication between the research team and the participants throughout the study’s duration. This lack of communication diminishes the connection between the participant and the study, which leads to participants forgetting that they are enrolled, ultimately resulting in drop out. It was also expressed that high commitment in a study was a barrier to completion. Participants stated that multi-session studies that are lengthy and far from their homes are not appealing over time. This commitment was said to become an inconvenience for participants, which contributed to their lack of retention. This barrier to retention was most relevant for participants experiencing health issues that constrained their ability to be away from home for long periods.

#### Individual-Level Facilitators

Many participants expressed that the most important facilitator for participating in research was the incentive offered, a theme that emerged strongly in focus group discussions and is detailed in Supplementary Figure S3, which presents the frequency of facilitators across focus groups. Participants stated that monetary compensation was one of the most effective ways to encourage their recruitment. For example, one participant stated “Money. Money’s always nice.” Participants also identified the study’s purpose and their expectations of its outcomes as key facilitators. Many individuals stated that if they perceived the study as beneficial for others, they would be more inclined to participate. Lastly, numerous participants indicated that they found studies aimed at improving their healthcare access to be more appealing. Participants stated that research has provided them with a more affordable option for receiving treatment for various health conditions compared to using insurance or seeking medical care elsewhere. A participant shared that participating in research “cuts back on my medical expenses, things that I would want to do, and when it comes to the money to do it, it’s just expensive, and if I go to the research, I wouldn’t have to pay for it.”

#### Interpersonal-Level Facilitators

Participants mentioned that communication and positive encounters with the study team encouraged participation. Key insights on this facilitator include:

“I’m much more likely to carry it out if I’ve had a positive experience with the coordinators and to extrapolate on that a little bit, I think that having coordinators that are very good at explaining what things are going to look like ahead of time, and I think just being kind and understanding and flexible and going the extra mile in terms of just customer service.”

Participants expressed that the flexibility and the convenience of the study, such as day and time, encouraged them to participate. Participants stated, “it’s not always convenient to go during a workday, and so, you know, having after hours options before work or after work options to complete study visits is always nice to have.”

#### Community-Level Facilitators

From a community-level perspective, flexibility and convenience in the study setting were important. Participants stated that studies held in a virtual setting were more desirable and better suited to their schedules. A participant stated that it would be helpful to have a “mechanism that you can do that like this, in a remote type of setting. Or, you know, complete visits online somehow through some type of online data capture.” Another participant stated, “phone visits or just online, completely. That’s super easy.”

#### Facilitators to Retention

Participants highlighted that their continued participation was often driven by personal and community factors. Many emphasized that they felt personally obligated to complete a study once they had agreed to start. This was linked to participants’ sense of fulfilling their commitment, their own experiences with a study topic, or being moved to complete a study to help family members and others in their community who might benefit from the findings. However, other participants felt that their retention in a study was driven by the commitment of time and travel. They expressed being more likely to complete a study when this commitment was low. Another facilitator was the availability of ongoing compensation, especially when the time and travel demands were high. Participants were more likely to remain consistent in their participation when they were compensated regularly. Additionally, they emphasized that a strong connection with the study team played a crucial role in their retention. Consistently interacting with the same team members and feeling valued as contributors to the study further motivated them to remain engaged. Participants also emphasized the importance of transparency from the study team regarding changes to the study and the factors influencing its progression. They expressed a desire for real-time access to study results, citing frustration with long delays before results were published or made public. Regular updates on study progress and findings were viewed as essential for maintaining their engagement.

### Addressing Barriers and Facilitators to Participation in Precision Health Research

Participants also shared their recommended strategies for overcoming identified barriers, as well as improving facilitators to encourage participation in precision health research (Supplementary Tables S4 and S5).

#### Recommendations for Addressing Barriers to Participation

According to the focus group sessions, general mistrust of researchers can be overcome through the use of marketing campaigns designed to reassure and educate potential participants about the safety of studies. The COVID-19 pandemic was brought up as a recent, significant contributor to this widespread mistrust. Participants mentioned that the plethora of information and misinformation that was shared during this time caused their trust in medical leaders to plummet. Rebuilding this trust between the public and the medical community could have a great impact on future enrollments rates. On the other hand, other participants stated that their relationship with medical leadership was positive. Additionally, for some participants, primary care providers were their most trusted source of health information and research opportunities. Therefore, by leveraging these doctor-patient relationships, researchers may be able to gain the trust of participants by ensuring they receive accurate and reliable information from their primary care providers.

The second most prevalent barrier was the time allotted for participation. Much like travel and geography, participants stated that they were often deterred from studies that were too much of a commitment. By this, they referred to studies requiring several activities, such as returning for multiple visits, performing various tasks, or attending sessions that were overly time-consuming. Participants suggested that researchers should be transparent about the commitment required by clearly stating how often participants are expected to engage (if multiple sessions are required) and the flexibility of scheduling. This factor did not yield many actionable suggestions, as it stemmed from broader societal conditions. Participants attributed the lack of time, or the inability to find an “extra hour” in the day, not to the study itself, but to the structure of their lifestyles and other high-priority commitments.

The third most noted barrier was travel and inconvenience. To address this, participants stated that it was essential to understand the underlying causes. Participants mentioned that financial obligations and childcare responsibilities were key contributing factors. This study distinguished this barrier from “time allotted to participation” by clarifying that time, as described by participants, referred to an inability to participate, a factor beyond researchers’ control. In contrast, inconvenience was identified as an accessibility issue, where participants might have an “extra hour” but found the means of participation too inconvenient. If participation were made more accessible, prolonged engagement and retention would likely increase. Participants suggested that sessions, when possible, be held in virtual settings at unconventional times such as evenings and weekends, to enhance accessibility. Other strategies included providing transportation through bus passes and ride-share services, opening multiple study sites to reduce transit times, and offering multiple time windows for patients to return when multiple sessions were required.

To combat the barriers of limited awareness of research opportunities and lack of communication with the study team, participants suggested that researchers take the initiative by actively recruiting potential participants and contacting them directly to determine their interests. Improved communication with the study team could provide participants with important information before the study begins. Across many focus group sessions, clear communication emerged as a key strategy for reducing barriers. Participants who were unaware of the study, concerned about privacy, unsure about the study’s purpose, or uncertain about eligibility requirements all benefited from communication with the study team. This proactive step may be effective for addressing many of the barriers mentioned. Furthermore, to maintain participant involvement, participants emphasized that regular engagement with the study team was key. They suggested that researchers send frequent reminders to those enrolled to help keep them engaged. Some participants indicated that text message systems would be a more efficient means of contact than email.

To alleviate participant frustration when they failed to meet eligibility requirements, participants suggested the implementation of a virtual pre-screening process to assess eligibility before attending in-person visits, thereby reducing barriers often associated with physical appointments (i.e., inconvenience of travel).

### Building on Facilitators to Participation

The most frequently noted facilitator to participation was the opportunity for a personal health benefit. Participants shared that their primary reason for participating in studies in the past was the belief that the study would help them understand or treat a personal health condition.

Compensation was also a common facilitator. Approximately 70% of sessions contained dialogue about compensation encouraging participation (Supplementary Figure S3). Participants suggested that to support retention, researchers should consider offering ongoing compensation throughout the study and ensure that compensation aligns with the level of effort required from participants.

Other facilitators, such as flexibility and convenience, communication with the study team, and awareness of the study, were closely tied to previously established barriers. The strategies proposed to enhance these facilitators align with those suggested for overcoming the corresponding barriers, highlighting the interconnected nature of participant needs and study design considerations.

## Discussion

This study used an exploratory qualitative approach to gain a deeper understanding of the factors that positively and negatively impact participation in precision health research among diverse residents of South Carolina, with the aim of reducing barriers in precision health studies. One of thes study’s strengths was the inclusion of a large proportion of Blacks or African Americans, double the state’s reported 26% Black population according to the 2020 US Census [15]. Key barriers to participation were identified across individual, interpersonal, and community levels. By examining upstream factors that contribute to exclusivity, such as certain social determinants, researchers can uncover the underlying causes of minimal participation and limited access to precision health benefits following care [3]. It is particularly crucial to include populations that have been historically underrepresented in research due to systemic barriers, such as discrimination in access to education and research opportunities, biased study designs, and a lack of targeted recruitment, to ensure diverse representation in studies [13].

At the individual level, the key barrier was not meeting eligibility requirements. Interpersonal barriers included communication with the study team, time allotted to participation, inconvenient meeting times, and mistrust. At the community level, barriers included a lack of awareness of research opportunities and the inconvenience of the study site location [8].

While our study reaffirmed several well-established barriers to research participation, it also revealed new findings shaped by public health and regional factors in South Carolina. Participants identified mistrust in healthcare providers and health institutions as the most predominant barrier (Supplementary Figure S2). This was exacerbated by the event of the COVID-19 pandemic through perceived inconsistencies in how information was communicated. Further, participants raised concerns over the protection of their sensitive health information and how it could be misused. This theme was brought out by the recent intensification of longstanding structural concerns in healthcare and research, which is in line with other established themes regarding mistrust, such as poor communication and lack of transparency [16, 17]. According to the responses provided in the focus group sessions, this lack of trust can be combatted by fostering connections within target communities and communicating the purpose of the study through trusted community leaders. To address these findings on a policy level, greater efforts are needed to strengthen data privacy protections, ensure transparency of data use, and build accountability in research practices, especially for populations that have been historically marginalized and are underrepresented in research [16].

Another key barrier was geographic inaccessibility in South Carolina, as participants from rural areas stated that research opportunities were often limited by travel constraints. This barrier was especially significant for participants with lupus and other illnesses, whose ability to participate in research activities depended heavily on the severity of their symptoms on a given day. According to participants, geographic inaccessibility was attributed to research opportunities being concentrated in more urban areas or to perceived notions that health providers in rural areas were not aware of or involved in precision health research. This structural limitation reinforces disparities in underrepresented groups and exposes a critical gap that must be addressed within these South Carolina communities. Given these findings, future researchers may benefit from actively engaging with these communities directly in rural settings and using alternative attendance methods to accommodate individuals with chronic illnesses. Policy-level solutions include building funding for local research infrastructure and telehealth participation through broadband services, mandating community-engaged recruitment and retention strategies, and expanding provider training to ensure rural communities have equitable access to precision health research [18].

Despite these barriers, several facilitators were stated. On an individual level, participants were motivated by personal health benefits, perceived contributions to others, and compensation. Our findings also revealed a key facilitator that offers valuable insights for researchers aiding the South Carolina lupus community. Participants with lupus expressed a strong desire to participate in precision health research if they believed the findings could help others living with lupus, particularly those who were newly diagnosed. This highlights the role of community benefit as a motivator for engagement in South Carolina and emphasizes that the framing of research participation in terms of advancing scientific understanding and supporting the larger lupus community may encourage research involvement. Interpersonally, the convenience of participation and proactive outreach by the study team encouraged involvement. At the community level, the key facilitator was participants’ awareness of the opportunities to participate. Researchers could enhance their efforts to promote research benefit and provide follow-up with participants, including sharing study findings and personal health data. This approach may help attract more participants from their target audience. This factor may attract those from lower socioeconomic status who need certain medication, but do not have the means to pay for it. This is especially true for those who are uninsured, as this status may interfere with their ability to access treatments tailored to their needs or follow the recommendations provided based on their conditions [3].

Participants shared recommendations to address barriers and enhance facilitators. Key recommendations at the individual level included researchers making connections with the community and effectively marketing their studies to better communicate study purpose and reduce mistrust. Leveraging the doctor-patient relationship was also recommended, as many participants reported relying on healthcare providers for research information. Interpersonal strategies included transparent communication about study requirements and levels of commitment, offering flexible meeting times, and providing frequent reminders to enrolled participants to maintain retention. At the community level, enhanced outreach efforts supported by proactive recruitment was reported helpful in addressing a lack of awareness about research opportunities. Participants also stated that virtual participation options should be used whenever possible to improve accessibility. Since the COVID-19 pandemic, conducting research in virtual or online environments has become increasingly common and demonstrated usefulness in producing practical results [19].

The suggestions in this study aimed to reduce barriers to research participation in future studies. Efforts like this, along with similar initiatives, are essential for addressing both objective and subjective challenges. A notable example is the government’s investment in the “All of Us” Research Program, a national initiative led by the National Institutes of Health (NIH). This program seeks to collect and analyze health data from at least one million individuals across the United States, with the goal of advancing health by developing more effective treatments and prevention strategies [20]. The “All of Us” program actively addresses participation barriers by prioritizing the recruitment of a diverse population, particularly those historically underrepresented in medical research [20]. Through technology-driven methods, enhanced accessibility, and strong community engagement, the program seeks to build trust, address concerns about data privacy, and confront past research abuses, ultimately aiming to remove obstacles that might prevent certain demographics from participating [21, 22].

While the study offers valuable insights about precision health research participation, it also has limitations. One limitation was the sample size, including the number of participants in each focus group. Facilitators hosted a total of 10 focus group sessions, with 8 sessions including between 1 and 3 participants. The smaller focus group sizes were necessary to accommodate participant availability and ensure inclusion of diverse perspectives. Saturation was assessed repeatedly throughout data collection and analysis. During this effort, the themes observed demonstrated redundancy, with groups reinforcing existing themes without introducing new concepts. While this limitation in generalizability is acknowledged due to a smaller sample size than the recommended standard of 6 to 8 participants, saturation was sufficiently achieved for the scope and aims of this exploratory study [23]. Additionally, we acknowledge the potential for researcher bias inherent in qualitative research and acknowledge this as a limitation of the study.

At the same time, these group sizes afforded relaxed conversations, which might decrease the possibility that a dominant personality can skew a discussion and lead to “groupthink” [24]. Although these participants provided great insights, the online nature of the sessions introduced minor technical challenges during the study. Some participants with external email addresses experienced difficulties accessing the session link, which occasionally required rescheduling or, in some instances, led to participant attrition. It is important to note that we did not have a focus group that included all participants who had been diagnosed with lupus. This opportunity may have granted new insight due to a difference in environment. Perhaps, this kind of group would have fostered a deeper conversation due to participants sharing similarities in their experiences. Finally, participants in this study were selected from a previous survey that included individuals from diverse backgrounds, both with and without prior research experience (425 people). For this study, we invited individuals from the survey who had expressed interest in joining a focus group (101 people). Of the 23 people who followed through with participation, 20 people indicated familiarity with the research process and/or the scientific method in the initial survey. Consequently, our findings may not be fully generalizable to broader populations without prior exposure to research. Additionally, it was later learned during focus groups that some participants had existing affiliations with the healthcare institution conducting the study. This limits the generalizability of our findings to individuals without these affiliations or research experience. Future research should prioritize recruiting more diverse samples, including individuals with no prior research experience, to enhance the applicability of findings across populations.

Based on these results, future intervention research should prioritize more inclusive recruitment strategies that address study accessibility for marginalized groups, including racial minorities and individuals living in rural areas. Furthermore, the study team’s outreach efforts prior to the study’s launch should be enhanced to effectively address post-pandemic mistrust. As noted, a proactive approach to recruitment is required to engage populations that may be unaware of the availability and reliability of current research opportunities. As research continues to evolve, studies should be willing to adopt more technology-driven methods to create accessible participation opportunities. This way, barriers related to time and travel can be effectively reduced. Although multiple barriers to participation exist, the strategies provided by these community members offer promising ways to address these challenges. Thus, the implementation of these strategies may strengthen participation in precision health research.

## Supplementary Material

Supplementary Materials

**Supplementary Information** The online version contains supplementary material available at https://doi.org/10.1007/s40615-026-02916-0.

## Figures and Tables

**Table 1:** Barriers and Facilitators to Participation in Research

	Barriers	Facilitators
Individual	• Eligibility requirement	• Personal health benefit • Perceived benefit to others • Perceived benefit to underrepresented groups • Compensation • Personal interest • Respect for institution • Advance lupus research
Interpersonal	• Lack of communication with the study team • Time allotted to participation (negative attitude towards length of sessions/commitment • Inconvenience (availability to attend) • Mistrust in the medical community	• Communication with study team • Flexibility and convenience (time)
Community	• Awareness of opportunities • Inconvenience (traveling to study site)	• Flexibility and convenience (setting) • Awareness of opportunity

## Data Availability

The data generated in the current study are available in the supplementary material, and available from the corresponding author upon reasonable request.
