# Supplementary material for: Factors Influencing Participation in Precision Health Research Among Diverse Individuals in South Carolina
Source: J Racial Ethn Health Disparities. Author manuscript; Available in PMC 2026 Sep 19. (PMC13589564; doi:10.1007/s40615-026-02916-0)
Supplement: Supplementary file 1 [file NIHMS2210170-supplement-Supplementary_Materials.docx]

**Supplementary Materials for**

Factors Influencing Participation and Retention in Precision Medicine Research: Insights from Participants in the Precision rEsearCh pArticipatioN (PECAN) Study

Bernardica L. Brown, Paula S. Ramos Lori Ann Ueberroth, Lee H. Moultrie, Marquetta L. Goodwine, Rachael J. Werner, Samiha T. Karim, Ingrid M. Wagner, Melissa A. Cunningham, Diane L. Kamen, Bethany J. Wolf, Caitlin G. Allen

| **File** | **Page** |
| --- | --- |
| Table S1. Participant demographics | 2 |
| Table S2. Barriers to participation and retention in research | 3 |
| Table S3. Facilitators to participation and retention in research | 7 |
| Table S4. Barriers and proposed strategies to enhance research participation and retention | 12 |
| Table S5. Facilitators and proposed strategies to enhance research participation and retention | 14 |
| Figure S1. Socioecological model | 16 |
| Figure S2. Frequency of barriers across focus groups | 17 |
| Figure S3. Frequency of facilitators across focus groups | 18 |
| Box S1. Focus Group Guide includes questions and prompts that facilitators used to guide the discussion | 19 |
| Box S2. Transcript summary provides a concise overview of the key points and themes that were extracted from the transcribed focus groups | 23 |

| **Table S1. Participant demographics**. Percentages calculated from 23 participants and rounded to the nearest tenth. | | |
| --- | --- | --- |
|  |  | **%** |
| Lupus | |  |
| Yes | | 6 (26%) |
| No | | 16 (70%) |
| Prefer not to answer | | 1 (4%) |
| Self-reported race | |  |
|  | African American | 10 (43%) |
|  | Two or More Races | 1 (4%) |
|  | White | 12 (52%) |
| Sex | |  |
|  | Female | 18 (78%) |
|  | Male | 5 (22%) |
| Age | |  |
|  | 18-25 | 1 (4%) |
|  | 26-35 | 4 (17%) |
|  | 36-45 | 6 (26%) |
|  | 46-55 | 6 (26%) |
|  | 56-65 | 4 (17%) |
|  | 66-75 | 2 (9%) |
|  | 76+ | 0 (0%) |
| Education | |  |
|  | Grades 12 or GED | 1 (4%) |
|  | College 1 to 3 years | 8 (35%) |
|  | College 4 years or more | 5 (22%) |
|  | Postgraduate | 8 (35%) |
|  | Prefer not to answer | 1 (4%) |
| Insurance coverage | | |
|  | Private | 17 (74%) |
|  | Medicare | 6 (26%) |
|  | Medicaid | 3 (13%) |
|  | Military | 1 (4%) |
|  | Other | 1 (4%) |
|  | None | 1 (4%) |
| Moderate (or higher) knowledge of: | | |
|  | Precision Medicine | 6 (26%) |
|  | Personalized Medicine | 12 (52%) |

| Table S2. Barriers to participation and retention in research. The table includes each barrier, summary of barriers’ impact on participation and retention, and key insights from focus group participants. | | | |
| --- | --- | --- | --- |
| **Level** | **Barrier** | **Summary** | **Quote** |
| **Individual** | Eligibility Requirements | Participants who found studies of interest were often deterred by unclear or poorly communicated eligibility criteria which disqualified them from moving forward. | “One of the barriers has been age. Well, they were looking for a certain segment of the population, but not until I was ready to apply did they say we are not seeking people of that particular age.” |
| **Interpersonal** | Lack of communication with the study team | Potential participants are less likely to be recruited if they lack sufficient information about the study, especially when personal health information is involved. | "Probably just knowing like again, just knowing like where those blood samples are being used and who's using it. Who has access and information if that information is provided, then yes" |
|  | Time allotted to participation (negative attitude towards length of sessions/ commitment) | Participants found high-commitment studies unfavorable and often inconvenient, describing them as time-consuming. They expressed a greater willingness to participate in studies with lower commitment requirements. | "I've been in studies where sometimes it’s time-consuming. It's too long.”  "Number of visits as well. I'm more likely to participate in a study where the commitment is low, AKA there's less of my time being used and the compensation or the outcome is higher." |
|  | Inconvenience (availability to attend) | Participants are less likely to be recruited and remain engaged in a study if its scheduling is inconvenient or does not align with their availability. | “You guys have to be on a certain schedule. So if things just don't line up with my schedule, I has been a barrier “  "Especially if I have to take a bunch of time off work, or if it's like really disruptive or something, then a lot less likely to do it. “  "I noticed a lot of people would like to do research, but they don't have the time. It's the distance and you know they have kids and they have making the choice of doing research or working. They work first. “  "You know, for some people, it's hard to find even that extra hour to dedicate to themselves or dedicate to participate in something like this." |
|  | Mistrust in the medical community | Participants had skepticism toward the medical community, especially in studies requiring health information disclosure. The COVID-19 pandemic further deepened this mistrust, as participants noted a decline in trust toward medical recommendations. African American participants highlighted historical injustices as a significant deterrent. | “People just don't trust automatically what their doctors recommending or what the medical community is saying.”  “Ever since COVID the trust level seemed like it has plummeted, especially in like certain age populations or kind of depending on what it is. “  "There's a lot of distrust of research in the black population, and especially so among males. “  "I could definitely see community-wise and not wanting to participate in research because historically it has not. They have some very public, some very catastrophic incidents that have involved black people and research studies. So initially I was a little apprehensive because of that.”  “Certain history can cause people to not want to participate in research in my community" |
| **Community** | Awareness of opportunities | Participants reported difficulty in finding studies. Those from rural areas expressed a lack of awareness and available resources to learn about ongoing research opportunities. | “You have to do deep searches and then their databases, like when you put in your information and they reach out to you, it's still about stuff that doesn't apply to you, so they gotta figure out a better system for that."  “Only one clinic here and it's a Franklin C federal. So, if you don't have a MUSC doctor per se, You're not gonna be Privy to these surveys and studies. “  "I think the barrier is just the general population not knowing about the studies that are going on." |
|  | Inconvenience (traveling to study site) | Participants cited distance to research sites as a barrier, noting that in-person studies are often inconvenient due to occupational, childcare, and transportation challenges. | “Depending on how I'm feeling, I may not be able to drive myself, so that has been a barrier at times “  “Distance has been a barrier at times when there were. You know, like if I didn't have a regular appointment and I live about an hour away.”  "The convenience of a study is very important to me. I'm probably not going out of my way to participate and one like if there's no compensation involved and it's not like, you know, an area that I normally go or a time that works for me. " |

| **Table S3. Facilitators to participation and retention in research**. The table includes each facilitator, summary of facilitators’ impact on participation and retention, and key insights from focus group participants. | | | |
| --- | --- | --- | --- |
| **Level** | **Facilitator** | **Summary** | **Quote** |
| **Individual** | Personal health benefit | Participants were motivated to enroll in studies they believed could aid in treating or better understanding their medical conditions. | “Cuts back on my medical expenses, things that I would want to do and when it comes to the money to do it, it's just expensive, and if I go to the research I wouldn't have to pay for it.”  “One of my eye medicine, if I had to pay for it, was $500. So when I went through the study and stuff, I didn't have to pay for the medicine. So it was better for me than going to study for I could get the medicine. “  “One particular study found a heart issue which answered so many questions I had about how I was feeling." |
|  | Perceived benefit to others | Participants stated they were more likely to enroll in studies if they believed their involvement would significantly benefit others. | “I've got six kids and five grandkids, and, you know, if one of them came down with scleroderma. And if I managed, you know, by participating in a study, I managed to get a cure or anything that gave any benefit to them a little bit quicker, than that would be very rewarding. “  “Just opportunity to be part of something way bigger than myself."  "I like to help my fellow man. We should be helping one another in any way possible." |
|  | Perceived benefit to underrepresented groups | Some participants, identifying as members of underrepresented groups, enrolled in studies to support their communities. They expressed concern for the well-being of these groups due to their lack of representation in research. | “So, and that's one of my concerns always is like, are we making sure that we're getting unrepresented folks in it especially with an illness like lupus that affects such a large-scale population of black women and Asian women. I think it's very important to make sure that they're fully represented.”  “One thing I know that a lot of African Americans don't participate choose not to participate, and I feel like we're underrepresented a lot of times when research is done. So typically you know I want to, I guess make sure that my voice is heard. Our voice is heard.” |
|  | Compensation | Compensation was a common facilitator, including both monetary rewards and the sense of contributing to others, particularly when they can witness the impact of their contribution. | "Money. Money's always nice.”  "I guess I would say the compensation is like a bonus.”  "The compensation really motivates me. I'd like to know that my time is worth the time to take.”  "Probably for a lot of people the reward is probably gonna be the best thing.”  “Letting them know that, yeah, this is what you're helping us do and actually giving them something they can see that, yeah, they are making a difference." |
|  | Personal interest | A personal interest in the research topic served as a facilitator to enrollment and retention. | "I think for me the first thing would be, is it something that I'm interested in." |
|  | Respect for institution | Participants who are familiar with and trust the institution conducting the research are more likely to participate. | “Any person that I know with a rheumatoid type of illness, I always tell them to go to MUSC just because of the wonderful experience that I've had personally and how having MUSC in my life has been extremely beneficial.”  "I don't really consider MUSC to the place where I would consider privacy concerns that much." |
|  | Advance lupus research | Participants with lupus stated that a prominent facilitator to participation in research is the opportunity to advance knowledge in the field, which helps inform researchers and supports others affected by the disease. | "I think for me I don't live in an area where there's a support group or where I can actively participate in and fundraising or the lupus foundation walkathons. Or you know those activities and so and this has happened to me and I hope it doesn't happen to a lot of people and lupus is not fun. And so it's, I guess in my own way a way of sharing my experience and my information. It was very hard for me when I was first diagnosed and I didn't know anything about lupus and so I wanna, I guess in my own way, try to facilitate for other people not to have such a hard experience.”  “The reason that I joined is because I felt like it could be, you know, if they could find out if the drug would help in those earlier cases, it could potentially help you know those early stage kind of lupus patients." |
| Interpersonal | Communication with study team | Participants indicated they are more likely to enroll and remain engaged in a study if they have a positive interaction with the recruiter and receive sufficient information about the study prior to enrollment. | “There is a customer service aspect or like a sales aspect of it as well. I'm probably more likely if I know that the PI is actually going to be participating in the research, that's very important to me too. I think it's important to me to see the person who is the PI, like actually participating.”  “I'm much more likely to carry it out if I've had a positive experience with the coordinators and to extrapolate on that a little bit, I think that. Having coordinators that are very good at explaining what things are going to look like ahead of time, and I think just being kind and understanding and flexible and kind of going the extra mile in terms of just customer service.”  "Just open communication. Just give him as much information as possible" |
|  | Flexibility and convenience (time) | Participants are motivated to participate if their schedules permit, particularly when meeting times are flexible, such as occurring at unconventional hours or days. | "it's not always convenient to go during a workday, and so, you know, having after hours options before work or after work options you know, complete study visits is always nice or to have. “ |
| Community | Flexibility and convenience (setting) | Participants are motivated to participate when the setting is convenient. Many participants preferred virtual settings. | “Mechanism that you can do that like this, you know in a remote type of setting. Or. You know, complete visits online somehow through some time of online data capture.”  “Phone visits or just online completely. That's super easy.”  "Online versus in person and those are usually the biggest things I think about." |
|  | Awareness of opportunity | Participants who are aware of the opportunity and the benefits of participating are more likely to enroll and remain engaged. | "I can speak for myself. A lot of people don't like to do research. And I think sometimes because they're not knowledgeable about how can help folks and help them at the same time.”  "The availability is a big thing for me. I'm not-I don't get all the time in the world to be searching for these things “  "I saw it advertised somewhere. It could have been in the newspaper. I want to find out what is particular to my DNA for my children, for my grandchildren and my ethnic background."  "I joined through Instagram ads. Also, still MUSC but Instagram ads is something that we see a lot.” |

| **Table S4. Barriers and proposed strategies to enhance research participation and retention.** The table includes each barrier, summary of barriers’ impact on participation and retention, and the proposed strategies. | | | |
| --- | --- | --- | --- |
| **Level** | **Barriers** | **Summary** | **Strategies To Overcome Barriers** |
| **Individual** | Eligibility Requirements | Participants who found studies of interest were often deterred by unclear or poorly communicated eligibility criteria which disqualified them from moving forward and discouraged future enrollment. | Implement a virtual pre-screening process that assesses eligibility before requiring attendance at in-person visits. |
| **Interpersonal** | Lack of communication with the study team | Potential participants are less likely to be recruited if they lack sufficient information about the study, especially when personal health information is involved. | Clearly outline the study details and reassure participants about the confidentiality of their information before initiating the study. |
|  | Time allotted to participation (negative attitude towards length of sessions/ commitment) | Participants found high-commitment studies unfavorable and often inconvenient, describing them as time-consuming. They expressed a greater willingness to participate in studies with lower commitment requirements. | Clearly outline the study details and the level of commitment required from participants. |
|  | Inconvenience (availability to attend) | Participants are less likely to be recruited and remain engaged in a study if scheduling is inconvenient or does not align with their availability. | Offer required sessions at non-traditional times, such as evenings and weekends. |
|  | Mistrust in the medical community | Participants had skepticism toward the medical community, especially in studies requiring health information disclosure. The COVID-19 pandemic further deepened this mistrust, as participants noted a decline in trust toward medical recommendations. African American participants highlighted historical injustices as a significant deterrent. | Build connections within target communities and communicate the study's purpose through trusted community leaders.  Start marketing campaigns designed to reassure and educate potential participants about study safety.  Form partnerships with primary care providers to build off the trust inherent in doctor-patient relationships. |
| Community | Awareness of opportunities | Participants reported difficulty in finding studies. Those from rural areas expressed a lack of awareness and available resources to learn about ongoing research opportunities. | Actively recruit participants by reaching out directly to assess their interests. |
|  | Inconvenience (setting) | Participants cited distance to research sites as a barrier, noting that in-person studies are often inconvenient due to occupational, childcare, and transportation challenges. | Offer virtual options.  Offer several meeting locations near participants' residential areas. |

| **Table S5. Facilitators and proposed strategies to enhance research participation and retention.** The table includes each facilitator, summary of facilitators’ impact on participation and retention, and the proposed strategies. | | | |
| --- | --- | --- | --- |
| **Level** | **Facilitator** | **Summary** | **Strategies To Reinforce Facilitators** |
| Individual | Personal health benefit | Participants were motivated to enroll in studies they believed could aid in treating or better understanding their medical conditions. | Improve efforts to promote personal health benefits of participation. |
|  | Perceived benefit to others | Participants stated they were more likely to enroll in studies if they believed their involvement would significantly benefit others. |  |
|  | Perceived benefit to underrepresented groups | Some participants, identifying as members of underrepresented groups, enrolled in studies to support their communities. They expressed concern for the well-being of these groups due to their lack of representation in research. |  |
|  | Compensation | Compensation was a common facilitator, including both monetary rewards and the sense of contributing to others, particularly when they can witness the impact of their contribution. | Offer ongoing compensation reflecting participants’ efforts. |
|  | Personal interest | A personal interest in the research topic served as a facilitator to enrollment and retention. |  |
|  | Respect for institution | Participants who are familiar with and trust the institution conducting the research are more likely to participate. |  |
|  | Advance lupus research | Participants with lupus stated that a prominent facilitator to participate in research is the opportunity to advance knowledge in the field, which helps inform researchers and supports others affected by the disease. |  |
| Interpersonal | Communication with study team | Participants indicated they are more likely to enroll and remain engaged in a study if they have a positive interaction with the recruiter and receive sufficient information about the study prior to enrollment. | Clearly outline study details, offer positive customer service, and reassure participants about the confidentiality of their information before initiating the study. |
|  | Flexibility and convenience (availability to attend) | Participants are motivated to participate if their schedules permit, particularly when meeting times are flexible. | Offer required sessions at non-traditional times, such as evenings and weekends. |
| Community | Flexibility and convenience (setting) | Participants are motivated to participate when the setting is convenient. | Offer virtual options.  Offer several meeting locations near participants' residential areas. |
|  | Awareness of opportunity | Participants who are aware of the opportunity and the benefits of participating are more likely to enroll and remain engaged. | Actively recruit participants by reaching out directly to assess their interests. |

**Figure S1**. Socioecological Model highlighting the key factors that influence decision-making processes.

**Figure S2.** Frequency of barriers across focus groups.

**Figure S3. Frequency of facilitators across focus groups.**

**Focus Group Guide**

Precision rEsearCh pArticipatioN (PECAN) study:

Qualitative Focus Group Guide

Thank you for taking the time to meet and discuss precision research today. We have a series of questions to help us understand your perspectives about precision health research. Please share your opinions candidly. You are welcome to skip questions or stop the interview at any time. Does anyone have any questions before we begin?

We would also like to record this focus group. Are you comfortable with us recording? *If yes, begin recording*

Please introduce yourself by sharing your preferred name. Note that we will not use your name or identifying information in any resulting reports.

*Thank you for introducing yourselves!*

Before we begin, I wanted to share definitions of some keywords so that we are all on the same page (*show on slides*).

*Genetic research: The study of genes (DNA instructions) and how traits are passed from one generation to the next.*

*Biological sample, specimen or biospecimen: Samples of material, such as urine, blood, tissue, cells, DNA, RNA, and protein from humans, animals, or plants.*

*Data: Any personal information, including health information (medical history, diagnoses, any laboratory or other test results, genetic data, therapies), demographics (e.g., income, education, marital status), insurance coverage, and lifestyle (e.g., use of tobacco or alcohol).*

*Precision health research: Involves collecting individual or personalized medicine data (medical and genetic data) and incorporating them with social, environmental, and behavioral data, to develop tailored approaches to prevent disease and promote health.*

*Now that we've covered the background of the focus group, let's move on to the interactive part of our session. Before we start,* does anyone have questions about these definitions or need clarification *about your role today?*

***Perceptions about research participation***

1. Have you previously participated in a research study?

a. If yes, can you describe the study and your experience participating? Why did you choose to participate?

b. If no, why did you not participate?

2. What is most important when deciding to participate in a research study?

3. What are your thoughts about barriers for participating in this type of research?

a. At the community level (awareness, culture, geography, transportation, partnership with research)

b. At the interpersonal level (attitudes, cost, time, confidentiality, security, privacy, peers’ discrimination, social support)

c. At the individual level (knowledge, beliefs, perceptions, income, insurance, education, age)

4. Are there specific facilitators for participating in this type of research?

a. At the community level (awareness, culture, geography, transportation, partnership with research)

b. At the interpersonal level (attitudes, cost, time, confidentiality, security, privacy, peers discrimination, social support)

c. At the individual level (knowledge, beliefs, perceptions, income, insurance, education, age)

5. How might culture or social norms in your community influence your decision to participate in research?

6. How do the people in your community (inner circle) feel about it?

***Retention of participants***

1. What motivates you to continue participating in research studies?

2. What strategies do you think would be most effective in ensuring that research participants remain actively engaged and motivated throughout the studies?

***Precision health research***

1. Before today, had you heard of precision medicine or precision health research?

a. Where did you hear about this?

2. How do you think precision health research is different from other research?

3. Do you feel that precision medicine is important for improving health outcomes? Why or why not?

4. How do you think precision health might impact health disparities?

***Opportunities to enhance research participation among racial and ethnic minorities***

1.What are ways you think participation in research studies could be improved?

a. At the community level (awareness, culture, geography, transportation, partnership with research)

b. At the interpersonal level (attitudes, cost, time, confidentiality, security, privacy, peers discrimination, social support)

*c.* At the individual level (knowledge, beliefs, perceptions, income, insurance, education, age)

2. Are there any social and structural changes that could help remove barriers to participating in research?

***Review of Survey Data***

Now, we would like to share some findings from the survey that you all participated. Is there anything you find interesting about these data? Anything you find surprising?

Most participants self-identified as African American or Black (45.5%) and white (40.3%).

Participants were 70.8% Female.

The proportion of participants familiar with precision medicine terminology was higher for females compared to males.

57.1% of the participants were younger than 46 years old, while 41.6% of the participants were 46 years old or older. The proportion of participants familiar with precision medicine terminology was higher for participants 46 or older compared to younger than 46

53.9% of the participants did not have a college degree, while 45.5% of the participants were college graduates.

When deciding to participate in a research study, Black individuals reported higher levels of importance for: (i) donating to non-profit vs for-profit researchers (P < 0.05), (ii) individual, familiar, or community benefits (P < 0.01), (iii) access, time, biospecimen, and compensation (P < 0.01), and (iv) privacy and confidentiality. They also placed high importance on community engagement in developing a policy on precision health research

**Three case studies**

Next, we would like to share three case studies and receive your responses about them. I will show the case study on the screen and read it aloud. We will take a few minutes to reflect individually and then will discuss as a group.

1. The All of Us Research Program is a federally funded initiative that is run by the National Institutes of Health. Carla heard about this program from her primary care doctor during an annual visit. She looked up the program online and saw that it is free to join but that the program would require she provide a saliva sample and access to her medical records. She decided not to participate in the research until she met again with her doctor to better understand how her medical records would be used in this study.

a. What do you think of Carla’s decision?

b. Would you have participated in this research? Why or why not?

c. What may have swayed you to participate?

2. John was doing research about lupus after his sister was diagnosed. He came across a new research study sponsored by MUSC that required giving a blood sample and responding to survey questions. He decided to enroll in the study because he thought it was important to help contribute to understanding his sister’s disease, even though he may not directly benefit.

a. What do you think of John’s decision?

b. Would you have participated in this research? Why or why not?

i. If not, describe the types of incentives that would make you more likely to participate.

c. What may have swayed you to participate?

3. Patricia’s family all decided to join a study that provides results for genetic risk for certain diseases. After hearing about her family’s experience, she was intrigued. She called the number on the study brochure and discovered that the project was sponsored by a for-profit company (industry). She researched the company online. She decided not to participate when she discovered that the company stored the data and that it may be used by researchers across the country.

a. What do you think of Patricia’s decision?

b. Would you have participated in this research? Why or why not?

i. If not, what are your concerns regarding the privacy and security of your health data?

c. What may have swayed you to participate?

Thank you for participating in this focus group. Before we finish, is there anything else anyone would like to share?

**Transcript Summary**

ID#:

Completed by:

Date:

Site:

Interviewer:

Interview Date:

Individual-Level Barrier

Code Definition: Description of personal challenges that may hinder participation in a study; may include knowledge, beliefs, perceptions, income, insurance, education, and age.

Question:

Answer:

Themes:

Interpersonal-Level Barrier

Code Definition: Description of interpersonal challenges/interactions with others that may hinder participation in a study; may include attitudes, cost, time, confidentiality, security, privacy, peer discrimination, and social support.

Question:

Answer:

Themes:

Community-Level Barrier

Code Definition: Description of challenges at the community level that may hinder participation in a study; may include awareness, culture, geography, transportation, and partnership with research.

Question:

Answer:

Themes:

Individual-Level Facilitator

Code Definition: Refers to any mention of personal factors or conditions that encourage or enable participation in the research study; may include knowledge, beliefs, perceptions, income, insurance, education, and age.

Question:

Answer:

Themes:

Interpersonal-Level Facilitator

Code Definition: Refers to any mention of relationships and social interactions that encourage or support participation in the research study; may include attitudes, cost, time, confidentiality, security, privacy, peers’ discrimination, and social support.

Question:

Answer:

Themes:

Community-Level Facilitator

Code Definition: Refers to any mention of community-based factors that encourage or support participation in the research study; may include awareness, culture, geography, transportation, and partnership with research.

Question:

Answer:

Themes:

Strategies for Reducing Barriers

Code Definition: Refers to any mention of strategies that can be used to reduce barriers to participating in research.

Question:

Answer:

Themes:

Retention Strategy

Code Definition: Refers to any mention of strategies that can be used to motivate participants to stay engaged throughout the study.

Question:

Answer:

Themes:

Retention Motivator

Code Definition: Describes the participant's rationale for remaining engaged in the study.

Question:

Answer:

Themes:

Prior Knowledge of Precision Medicine

Code Definition: Refers to any mention of previous awareness/understanding/exposure to precision medicine before their involvement in the study; may include personalized medical approaches, genetic testing, and/or tailored treatments.

Question:

Answer:

Themes:

Source of Precision Medicine Information

Code Definition: Description of where participants obtain information about precision medicine.

Question:

Answer:

Themes:

Precision Medicine and Health Outcomes

Code Definition: Refers to discussion of the impact of precision medicine on health outcomes and how precision medicine has/will affect health, quality of life, and/or treatment.

Question:

Answer:

Themes:

Precision Medicine and Health Disparities

Code Definition: Refers to discussion of the relationship between precision medicine and health disparities.

Question:

Answer:

Themes:

Case Study #1 Barriers

Code Definition: Description of personal challenges that may hinder participation in a study; may include knowledge, beliefs, perceptions, income, insurance, education, and age. Description of interpersonal challenges/interactions with others that may hinder participation in a study; may include attitudes, cost, time, confidentiality, security, privacy, peers’ discrimination, and social support. Description of challenges at the community level that may hinder participation in a study; may include awareness, culture, geography, transportation, and partnership with research.

Question:

Answer:

Themes:

Case Study #1 Facilitators

Code Definition: Refers to any mention of personal factors or conditions that encourage or enable participation in the research study; may include knowledge, beliefs, perceptions, income, insurance, education, and age. Refers to any mention of relationships and social interactions that encourage or support participation in the research study; may include attitudes, cost, time, confidentiality, security, privacy, peers’ discrimination, and social support. Refers to any mention of relationships and social interactions that encourage or support participation in the research study; may include attitudes, cost, time, confidentiality, security, privacy, peers’ discrimination, and social support.

Question:

Answer:

Themes:

Case Study #2 Barriers

Code Definition: Description of personal challenges that may hinder participation in a study; may include knowledge, beliefs, perceptions, income, insurance, education, and age. Description of interpersonal challenges/interactions with others that may hinder participation in a study; may include attitudes, cost, time, confidentiality, security, privacy, peers’ discrimination, and social support. Description of challenges at the community level that may hinder participation in a study; may include awareness, culture, geography, transportation, and partnership with research.

Question:

Answer:

Themes:

Case Study #2 Facilitators

Code Definition: Refers to any mention of personal factors or conditions that encourage or enable participation in the research study; may include knowledge, beliefs, perceptions, income, insurance, education, and age. Refers to any mention of relationships and social interactions that encourage or support participation in the research study; may include attitudes, cost, time, confidentiality, security, privacy, peers’ discrimination, and social support. Refers to any mention of relationships and social interactions that encourage or support participation in the research study; may include attitudes, cost, time, confidentiality, security, privacy, peers’ discrimination, and social support.

Question:

Answer:

Themes:

Case Study #3 Barriers

Code Definition: Description of personal challenges that may hinder participation in a study; may include knowledge, beliefs, perceptions, income, insurance, education, and age. Description of interpersonal challenges/interactions with others that may hinder participation in a study; may include attitudes, cost, time, confidentiality, security, privacy, peers’ discrimination, and social support. Description of challenges at the community level that may hinder participation in a study; may include awareness, culture, geography, transportation, and partnership with research.

Question:

Answer:

Themes:

Case Study #3 Facilitators

Code Definition: Refers to any mention of personal factors or conditions that encourage or enable participation in the research study; may include knowledge, beliefs, perceptions, income, insurance, education, and age. Refers to any mention of relationships and social interactions that encourage or support participation in the research study; may include attitudes, cost, time, confidentiality, security, privacy, peers’ discrimination, and social support. Refers to any mention of relationships and social interactions that encourage or support participation in the research study; may include attitudes, cost, time, confidentiality, security, privacy, peers’ discrimination, and social support.

Question:

Answer:

Themes:
